# Observational Study on the Occurrence of Muscle Spindles in Human Digastric and Mylohyoideus Muscles

**DOI:** 10.1155/2014/294263

**Published:** 2014-07-16

**Authors:** Daniele Saverino, Amleto De Santanna, Rita Simone, Stefano Cervioni, Erik Cattrysse, Marco Testa

**Affiliations:** ^1^Dipartimento di Medicina Sperimentale, Sezione di Anatomia Umana, Università degli Studi Genova, 16132 Genova, Italy; ^2^Dipartimento di Medicina Sperimentale, Sezione di Istologia, Università degli Studi Genova, 16132 Genova, Italy; ^3^The Feinstein Institute for Medical Research, North Shore-LIJ Health System, Manhasset, NY 11030, USA; ^4^Afdeling Experimentele Anatomie, Vrije Universiteit Brussel, 1050 Brussels, Belgium; ^5^Dipartimento di Neuroscienze, Riabilitazione, Oftalmologia, Genetica e Scienze Materno Infantili, Campus Universitario di Savona, Università degli Studi Genova, 17100 Savona, Italy

## Abstract

Although the occurrence of muscle spindles (MS) is quite high in most skeletal muscles of humans, few MS, or even absence, have been reported in digastric and mylohyoideus muscles. Even if this condition is generally accepted and quoted in many papers and books, observational studies are scarce and based on histological sections of a low number of specimens. The aim of the present study is to confirm previous data, assessing MS number in a sample of digastric and mylohyoideus muscles. We investigated 11 digastric and 6 mylohyoideus muscles from 13 donors. Muscle samples were embedded in paraffin wax, cross-sectioned in a rostrocaudal direction, and stained using haematoxylin-eosin. A mean of 5.1 ± 1.1 (range 3–7) MS was found in digastric muscles and mean of 0.5 ± 0.8 (range 0–2) in mylohyoideus muscles. A significant difference (*P* < 0.001) was found with the control sample, confirming the correctness of the histological procedure. Our results support general belief that the absolute number of spindles is sparse in digastric and mylohyoideus muscles. External forces, such as food resistance during chewing or gravity, do not counteract jaw-opening muscles. It is conceivable that this condition gives them a limited proprioceptive importance and a reduced need for having specific receptors as MS.

## 1. Introduction

Muscle spindles (MS) were recognized as specialized entities of the skeletal (somatic) musculature of vertebrates since the early 1860s [1]. The morphology and function of MS have been widely described in different species, including humans [2–4]. MS are very complex sense organs that provide critical information for the function of motor control. MS consist of a group of differentiated muscle fibers, called intrafusal fibers, surrounded over the central part by a fusiform capsule filled with a highly viscous fluid. The intrafusal muscle fibers are noncontractile in their central portion, under the capsule's juxtequatorial protuberance. MS lie in parallel to the muscle-fibers and in series to the elastic elements, contributing to the complex and functionally partitioned muscle's architecture. Spindle deformations, related to the variation of muscle's length, are monitored by specialized sensory fibers (types Ia and II), which contact the intrafusal fibers only in the multinucleated (and thus noncontractile) region under the equatorial region of the capsule [5]. There are several evidences that spindles in skeletal muscles are part of a complex functional system. They possess multiple roles such as generating antigravity thrust during quiet upright stance, timing of locomotor phases, correcting for muscle nonlinearities, compensating for muscle fatigue, determining synergy formation, and modulating plasticity and motor learning [5, 6].

The task-dependent fusimotor inputs codetermine spindle activity and, in turn, the complex central connectivity codetermines the functional use of spindle afferent signals. In addition, the motor neurons innervating the contractile peripheral part of the MS maintain the firing of spindle afferents when the contraction of extrafusal muscles' fibers shortens the muscle.

In mammalians, the number of MS in a muscle seems to be related to its function and widely varies from one muscle to the other [2, 4]. The presence of MS in muscles involved in fine movements, as in small muscles of the distal extremities, is particularly high [7]. This suggests that smaller muscles show a higher spindle density if compared to larger ones [2, 8, 9]. Some exceptions were shown at the level of orofacial area. MS seem to be absent in anterior digastric muscle and mylohyoid of cats [10] and in lateral pterygoid muscles, anterior digastric muscle, posterior digastric muscle, and stylohyoid of rats [11].

In humans, data on the number and density of spindles in suprahyoid muscles are scarce and quite old and most of them were obtained from the analyses of a small number of specimens [9]. Lennartsson [12] analyzed anterior belly of digastric muscles from five subjects and confirmed the rareness of these receptors. Though studies on this topic are very rare and old, there is a general consensus that MS are almost absent in suprahyoid muscles. The aim of the present study is to investigate the presence and density of MS in a sample of human digastric and mylohyoideus muscles, in order to confirm and eventually reinforce the data available in literature.

## 2. Materials and Methods

### 2.1. Study Design

The specimens were collected from cadavers of 13 donors (60–90 years old, 8 men and 5 women) and used for lessons of anatomy at the Experimental Anatomy Department of the Vrije Universiteit Brussel. Muscle samples were consecutively selected, depending on their good morphological aspect and the possibility to take them out* in toto*. Based on these criteria, 11 anterior and posterior bellies of digastric muscles from 10 donors, in one subject, where we had the possibility to analyze left and right muscles, and 6 mylohyoideus muscles, from six different donors, were included for the analysis. To confirm the correctness of the histological procedure, a control sample of 4 plantaris muscles, that are known to be rich of MS, was analyzed. Subjects donated their body for research and education. Neither personal history nor medical antecedents of donors were available. A written informed consent was obtained from all the relatives of the donors and all the samples were obtained following the ethical guidelines of the most recent Declaration of Helsinki (Edinburg 2000). The study received the approval from the ethical commission of the Brussels Academic Hospital, associated with Vrije Universiteit Brussel (B.U.N. 143201317580).

Immediately after removal, muscle samples were washed with cold PBS to take out all blood and then fixed with 20% paraformaldehyde in PBS buffer and embedded in paraffin wax. Cross-sections were cut in a rostrocaudal direction and transferred into 2% gelatinized glass slides for histologic staining. Sampled sections (5 *μ*m thick) were stained using haematoxylin-eosin and then examined, whereas equidistant intermediate sections were left unstained. Sections at 1 mm intervals were considered appropriate on the basis of a pilot study, in which muscle tissues from 4 plantaris muscles control samples were sectioned and the spindle length was determined.

### 2.2. Method for Determining Spindle Distribution

We used Leica microscope (Leica Microsystems, Milano, Italy), calibrated to a digitizing system, and Leica FireCam 1.9.2 software (Leica Microsystems, Milano, Italy). Muscle section perimeters were traced using an ×4 objective and the location of all identified MS plotted using an ×10 objective for stained sections. The distribution of MS in the plotted muscle sections was determined using a rectangular grid positioned over each plotted section.

Count spindle numbers were determined following the stereologic method [13, 14]. In brief, one or more MS appearing in consecutive serial sections have to be counted only once, in order to avoid duplicate counting. It was decided to count spindles only when they appeared from one 5 *μ*m section to the next in the rostrocaudal series of sections. Only spindles that appeared in one section were counted in accordance with stereologic practice.

Finally, spindle density was evaluated as the spindle number per gram of wet muscle tissue for each muscle sample. The digastric muscle had a mean tissue weight greater than the mylohyoideus (7.2 versus 5.4 g).

### 2.3. Statistical Analysis

Muscle spindle counts were used to test differences among digastric, mylohyoideus, and plantaris muscles. As muscle groups are of different size, arithmetic convenience causes us to choose the sum of ranks for the smaller group. Thus, an unpaired *t*-test with Welch's correction [13] was used to account for heterogeneity of variances in study groups. Differences were considered statistically significant at a *P* value <0.05. Statistical analysis was performed by Graph Pad Statistical software, version 6.

## 3. Results

### 3.1. Muscle Spindles Analysis

**Figure 1:**
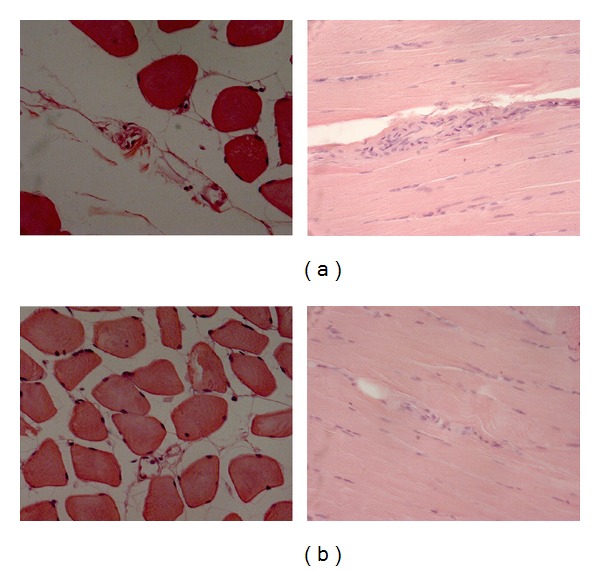
As an example muscle spindles in digastric (a) and mylohyoideus (b) muscles are shown.

We analyzed several sections of digastric and mylohyoideus muscles from different donors (as an example, see Figures 1(a) and 1(b)). As reported in Table 1, a very small number of spindles were found in digastric (mean 5.1, standard deviation 1.1; range 3–7) and mylohyoideus (mean 0.5, standard deviation 0.8; range 0–2) muscles. All MS were present in the anterior belly of digastric muscle, whereas none was found in the posterior belly. In one donor, where we had the possibility to investigate left and right digastric muscle, an almost equal number of spindles were found (5 and 6 MS, resp.). Mylohyoideus muscles seem to be characterised by a much lower number of spindles (Table 1).

**Figure 2:**
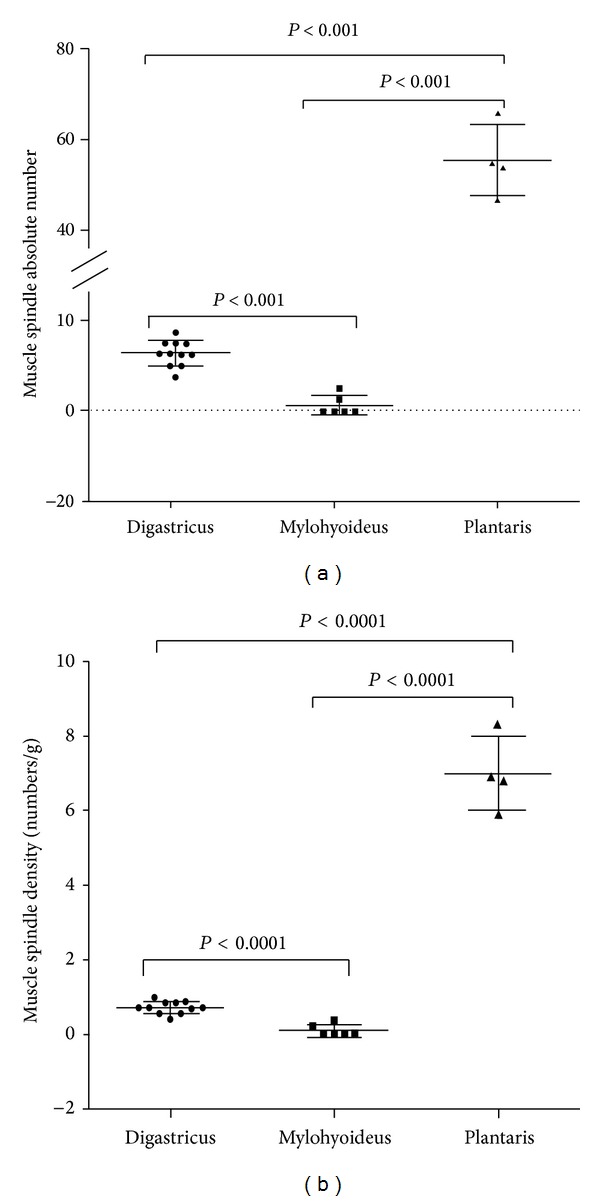
Muscle spindles differences among digastric, mylohyoideus, and plantaris muscles expressed as absolute numbers (a) and density (b). Bars represent mean values and standard deviation. *P* values calculated by unpaired *t*-test with Welch's correction are shown.

A high number of MS were identified in the control sample of plantaris muscles (mean 55.5, standard deviation 7.8; range 47–66). Based on the unpaired *t*-test with Welch's correction, the differences in MS absolute numbers between digastric and mylohyoideus muscles were statistically significant (*P* < 0.001). Similar results were observed comparing digastric muscle (*P* < 0.001), and mylohyoideus to plantaris (*P* < 0.001) (Figure 2(a)).

The measurement of spindle density was 0.7/g (range 0.4–9.7, standard deviation 0.2) for digastric muscle and 0.1/g (range 0–0.4, standard deviation 0.2) for mylohyoideus. Spindles density of plantaris muscles was 7.9/g (mean tissue weight of 19.4 g; range 5.9–8.3; standard deviation 1.0).

Unpaired *t*-test with Welch's correction showed a significant difference in spindle density between digastric muscle and mylohyoideus (*P* < 0.001) (Figure 2(b)) and between digastric muscle and mylohyoideus with plantaris muscles (*P* < 0.001 for both comparison) (Figure 2(b)).

**Figure 3:**
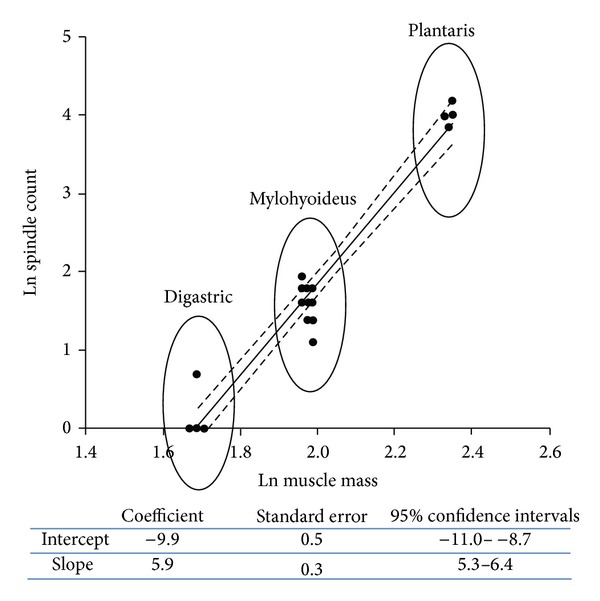
Scatter plots of logarithmically transformed spindle counts against muscle mass for the complete sample of human muscles. The insert shows a summary of the statistical analysis.

Finally, we performed a new analysis by logarithmic transformation of the data, followed by regression analysis of the logarithm of spindle number against the logarithm of muscle mass [15]. In fact, as Banks shows, despite the common use of spindle density in comparative studies, it has never been demonstrated that muscle mass is an appropriate reference for spindle number. A scatter plot of the logarithmically transformed data is provided in Figure 3, within a summary of the statistical analysis. As can be depicted from Figure 3, also by this method, in agreement to spindle density, there is a decrease in relative abundance in digastric muscles and mylohyoideus compared to plantaris ones.

## 4. Discussion

The current study examined numbers and density of spindle cells in a cadaveric sample of digastric and mylohyoideus muscles. This investigation was performed with cadaveric preparation because, to obtain unbiased estimates of spindle characteristics, it is preferable to assess suprahyoid muscles* en mass* rather than partial samples obtained by biopsies, during surgery. Moreover, previous studies have verified that spindles are resilient to change and that fiber type determinations are reproducible in postmortem tissues [13].

While muscle spindles are generally abundant in human skeletal muscles [9, 15], there is a consensus about lack or paucity of spindle cells in the suprahyoid muscles [9, 12, 15, 16]. This commonly accepted statement, beared by few, generally old, experimental studies and conducted on a very limited number of specimens [9, 12, 15, 16], was confirmed by our results. Lennartsson [12] identified 12 spindles in 10 anterior bellies of digastric muscle; of them 8 were retrieved in the same subject and one subject was completely devoid of spindles.

We found a mean of 5.1 receptors in a sample of 11* in toto *digastric muscles taken out from 10 subjects, while Lennartsson [12] reported a mean of 1.2 receptors in his sample of 5 subjects. In our sample, spindles occurred in every muscle, with a mode of 6 and a median of 5, while they were, respectively, 0 and 0.5 in the study of Lennartson [12].

In the literature of the last 50 years, out of our study, only Voss [9] analyzed the number of spindles in the mylohyoideus muscle. In his unique sample, he did not find any spindles. This result is consistent with our analysis of 6 samples that revealed a mean occurrence of 0.5, with a mode and a median of 0 (Table 2).

With respect to the spindle density, several points remain to be clearly defined. It could be envisaged that the correlation between spindle abundance and muscle mass may be true for postural muscles, but high spindle density was also found in some intrinsic hand muscles [15].

MS, altogether with Golgi tendon organs, by their proprioceptive information, contribute to the control of kinematic and dynamic variables. Thus, the abundance and intramuscular location of spindles seems to be somehow related to their physiological functions [5, 6].

So far, few studies provide data sufficient to deal with the distribution of spindles [7, 9, 15]. In these studies, the presence of spindles in skeletal muscles has been quantified by spindle density. The prototype of these studies provides data from 138 human muscles (based mostly on human muscles of newborn and infants, analyzed from 1937 to 1971) [9].

Boyd-Clark et al. [13] conducted an analysis of the number of MS in relation to muscle mass in mammalians (mouse, rat, guinea-pig, cat, and human) skeletal muscles. Significant differences in relative spindle abundance were found: the greatest abundance was in axial muscles, including those involved in head position, whereas the smaller was in muscles of the shoulders. The author found no differences between large and small muscles operating in parallel, or between antigravity and nonantigravity muscles.

Notably, a linear correlation between muscle mass and spindle content has never been demonstrated [17], suggesting that an additional anatomical feature of similar quantitative nature may exist. Such a feature should be related to the oxidative angiotype, that is, the vessel tree supplying oxidative muscle [5, 18–20]. This supports the higher presence of spindles in axial muscles compared to locomotor ones, due to the higher percentage of oxidative fibers in postural muscles [5, 18–20]. However, there are still some debates as MS should be considered not only in relation to a simplistic virtual “muscle length” machinery but also in a wider contexts of complex functional systems. Thus, it seems that MS would be related to multiple roles in regulating and coordinating muscle functions [6]. In addition, small muscles, required for fine motor control, have large spindle densities, whereas those recruited for rapid movement need comparatively a lower spindle density [21, 22].

The number of MS in muscle seems to depend on specific function. Kubota and Masegi [16] evidenced that, among human jaw muscles, only the lateral pterygoid muscle, involved in jaw opening as suprahyoid muscles, contains very few MS, while a relevant number of them are present in temporalis, masseter, and medial pterygoid muscles.

While the jaw closer muscles need to develop higher and well-controlled levels of force, the jaw opener muscles are designed to displace the jaw with maximal velocity [23]. In fact, muscles like masseters have to regulate precisely their force during mastication, depending on the hardness of the food or on the need to manage an object kept between teeth, while suprahyoid muscles mainly intervene to accelerate the mouth opening, facilitated by gravity and by diminishing of the jaw closer muscle tone.

Paucity or absence of spindle in the digastric and other jaw opener muscles is additionally supported by the fact that they present little or no reflex activity [24, 25], while an evident reflex response has been shown in the masseter, and other jaw-closing muscles.

Although some authors claimed that a stretch reflex cannot be obtained from the digastricus [26], nevertheless, a reflex activity seems not completely absent in the digastric muscle. This was elegantly demonstrated by Ostry et al. [27] with a jaw-unloading methodology of assessment and it is justified by the, even limited, presence of spindles in the digastric muscles, as we showed in our investigation.

From a functional perspective, it is likely that control of the digastric muscles is part of a complex control that also coordinates the activity of muscles attached to the hyoid bone and possibly digastric muscle activity is subject to influences from sensors in muscles connected to the hyoid bone [28].

While paucity or absence of MS in some suprahyoid muscles still remains an enigma, few speculations on this condition could be purposed.

The lack of MS in suprahyoid muscles could be seen as an advantage in case of spasticity associated to upper motor neuron syndrome. In such a condition, if MS were present within the suprahyoid muscles, a rapid change of length of this muscle, that is, due to passage of the food bolus, head movements, and so forth, will lead to abnormal increase of tension in the throat, larynx, and mouth, hindering activities essential for survival like feeding, breathing, and/or phonation.

The need for MS in jaw opener muscles is reduced by their usual relationship with external forces, such as gravity. Opening of the mouth occurs in gravity favour and can be mainly controlled by the variations of jaw closer muscle tone. Additionally, jaw opener muscles, are almost never subjected to condition of stretching, because of the limit, given to mouth closing, by the teeth contact.

*Study Limitation*. This study presents some limitations. First of all we had to collect muscles samples when eligible from the available cadavers used for anatomy lessons at the University; therefore, we were not able to set up a more representative sample, balanced among males and females, with both right and left muscles taken out from the same cadaver. Second, our sample is still small, even if significantly larger then samples used by the nowadays published studies. Third, the control group was chosen only to control the correctness of the procedure and it is composed by a very limited number of specimens. Even though the difference in number of spindles is highly evident, it would have been better, for statistical analysis, to build up a control group comparable, in number, to the experimental one.

## Figures and Tables

**Table 1:** Absolute number of muscle spindles in selected suprahyoid muscles (digastric and mylohyoideus) and in controls (plantaris).

Digastric (specimen number/side)	*N*	Mylohyoideus (specimen number/side)	*N*	Plantaris (specimen number/side)	*N*
21 L	5	19 R	0	24 L	55
21 R	6	21 L	1	28 L	47
23 L	4	41 R	0	39 R	54
22 L	5	39 R	2	39 L	66
50 R	3	51 R	0		
29 R	6	29 R	0		
41 R	7				
39 R	5				
43 R	4				
19 R	5				
49 R	6				

Average number	5.1	Average number	0.5	Average number	55.5

**Table 2:** A comparison of results from revised study and the present study.

	Number of analyzed muscles (*n*)	Average spindle numbers (when *n* > 2)	Reference
Digastricus	11	5.1	This study
	2	7.5	[9]
	10	1.2	[12]
	2	0	[16]
	2	7.5	[15]

Mylohyoideus	6	0.5	This study
	1	0	[9]

## References

[1] Matthews PBC (1972). *Mammalian Muscle Receptors and Their Central Action*.

[2] Barker D, Hunt CC (1974). The morphology of muscle receptors. *Handbook of Sensory Physiology. III/2 Muscle Receptors*.

[3] Maier A (1992). The avian muscle spindle. *Anatomy and Embryology*.

[4] Barker D, Banks RW, Engel A, Franzini-Armstrong C (1994). The muscle spindle. *Myology*.

[5] Kokkorogiannis T (2004). Somatic and intramuscular distribution of muscle spindles and their relation to muscular angiotypes. *Journal of Theoretical Biology*.

[6] Windhorst U (2008). Muscle spindles are multi-functional. *Brain Research Bulletin*.

[7] Botterman B, Binder M, Stuart D (1978). Functional anatomy of the association between motor units and muscle receptors. *American Zoologist*.

[8] Hosokawa H (1961). Proprioceptive innervation of striated muscles in the territory of cranial nerves. *Texas Reports on Biology and Medicine*.

[9] Voss H (1971). Tabell der absoluten and relativen Muskelspindlezahlen der menschlichen Skelettmuskulatur. *Anatomischer Anzeiger*.

[10] Morimoto T, Inoue H, Kawamura Y (1982). Diameter spectra of sensory and motor fibers in nerves to jaw-closing and jaw-opening muscles in the rat. *Japanese Journal of Physiology*.

[11] Maier A (1979). Occurrence and distribution of muscle spindles in masticatory and suprahyoid muscles of the rat. *the American Journal of Anatomy*.

[12] Lennartsson B (1979). Muscle spindles in the human anterior digastric muscle. *Acta Odontologica Scandinavica*.

[13] Boyd-Clark LC, Briggs CA, Galea MP (2002). Muscle spindle distribution, morphology, and density in longus colli and multifidus muscles of the cervical spine. *Spine*.

[14] Gundersen HJG, Bagger P, Bendtsen TF (1988). The new stereological tools: disector, fractionator, mucleator and point sampled intercepts and their use in pathological research and diagnosis. *APMIS*.

[15] Banks RW (2006). An allometric analysis of the number of muscle spindles in mammalian skeletal muscles. *Journal of Anatomy*.

[16] Kubota K, Masegi T (1977). Muscle spindle supply to the human jaw muscle. *Journal of Dental Research*.

[17] Banks R, Stacey M (1987). Quantitative studies on mammalian muscle spindles. *Mechanoreceptors. Development, Structure and Function*.

[18] Conley MS, Meyer RA, Bloomberg JJ, Feeback DL, Dudley GA (1995). Noninvasive analysis of human neck muscle function. *Spine*.

[19] Roy RR, Ishihara A (1997). Overview: functional implications of the design of skeletal muscles. *Acta Anatomica*.

[20] Vasavada AN, Li S, Delp SL (1998). Influence of muscle morphometry and moment arms on the moment-generating capacity of human neck muscles. *Spine*.

[21] Smith RD, Marcarian HQ (1967). The neuromuscular spindles of the lateral pterygoid muscle. *Anatomischer Anzeiger*.

[22] Peck D, Buxton DF, Nitz A (1984). A comparison of spindle concentrations in large and small muscles acting in parallel combinations. *Journal of Morphology*.

[23] van Eijden TM, Korfage JA, Brugman P (1997). Architecture of the human jaw-closing and jaw-opening muscles. *Anatomical Record*.

[24] Goldberg LJ, Anderson DJ, Mathews B (1976). Changes in the excitability of elevator and depressor motoneurons produced by stimulation of intra-oral nerves. *Mastication*.

[25] Miles TS, Flavel SC, Nordstrom MA (2004). Stretch reflexes in the human masticatory muscles: a brief review and a new functional role. *Human Movement Science*.

[26] Wyke BD (1974). Neuromuscular mechanisms influencing mandibular posture: a neurologist's review of current concepts. *Journal of Dentistry*.

[27] Ostry DJ, Gribble PL, Levin MF, Feldman AG (1997). Phasic and tonic stretch reflexes in muscles with few muscle spindles: human jaw-opener muscles. *Experimental Brain Research*.

[28] Abbink JH, van der Bilt A, Bosman F, van der Glas HW (1998). A comparison of jaw-opener and jaw-closer muscle activity in humans to overcome an external force counteracting jaw movement. *Experimental Brain Research*.

